# Lucid dreaming of a prior virtual-reality experience with ego-transcendent qualities: a proof-of-concept study

**DOI:** 10.1093/nc/niaf017

**Published:** 2025-08-05

**Authors:** Daniel J Morris, D Blaise Elliott, S Gabriela Torres-Platas, Justin Wall, Ema Demšar, Karen R Konkoly, Emily Rosman, Marcia Grabowecky, David R Glowacki, Ken A Paller

**Affiliations:** Department of Psychology, Northwestern University, 2029 Sheridan Road, Evanston, IL 60208, United States; Department of Psychology, Northwestern University, 2029 Sheridan Road, Evanston, IL 60208, United States; Department of Psychology, Northwestern University, 2029 Sheridan Road, Evanston, IL 60208, United States; Milarepa Center, 6231 Paul Boone Rd, Tallassee, TN 37878, United States; Monash Centre for Consciousness and Contemplative Studies, Monash University, 29 Ancora Imparo Way, Clayton VIC 3168, Australia; Department of Psychology, Northwestern University, 2029 Sheridan Road, Evanston, IL 60208, United States; Department of Psychology, Northwestern University, 2029 Sheridan Road, Evanston, IL 60208, United States; Department of Psychology, Northwestern University, 2029 Sheridan Road, Evanston, IL 60208, United States; Intangible Realities Laboratory, CiTIUS—Centro Singular de Investigación en Tecnoloxías Intelixentes da USC, Rúa de Jenaro de la Fuente Domínguez, 15782 Santiago de Compostela, A Coruña, Spain; Department of Psychology, Northwestern University, 2029 Sheridan Road, Evanston, IL 60208, United States

**Keywords:** sleep and dreaming, self, metacognition, embodiment

## Abstract

The immersive environments of virtual reality (VR) have potential to engender a vast range of experiences. Although participants recognize these experiences as artificial, the consequences can still be profound. Compared to VR, lucid dreams—characterized by awareness that one is dreaming—potentially allow for even more expansive explorations of immersive multisensory experience. Furthermore, lucid dreaming could conceivably enhance the impact of a prior VR experience, producing more profound effects than the VR experience alone. As an initial step along those lines, we attempted to induce lucid dreams about a VR experience called *Ripple*, with the goal of documenting the impact of the combination. In prior research, *Ripple* by itself was shown to reduce self-other boundaries and enhance interconnectedness. We recruited four frequent lucid dreamers to experience *Ripple* on two occasions, followed by an overnight session with sounds from *Ripple* presented quietly during polysomnographically verified rapid eye movement (REM) sleep. Three participants experienced lucid dreams about *Ripple* that night, and all four reported dreams containing elements of *Ripple*. The lucid dreams were validated in real time by physiological signals from the dreamers to indicate their concurrent experience of lucidity in the dream, followed by signals of dreaming about the VR experience. On this basis, we can confirm that it was possible in these circumstances for people to have lucid dreams recapitulating elements of the prior VR experience. Our findings also showcase how the synergistic combination of VR and lucid dreaming could be strongly beneficial.

## Introduction

Researchers across psychology, religious studies, neuroscience, and applied sciences have taken interest in how a sense of human interconnectedness can be deepened via self-transcendent experiences (STEs), defined as mental states that transcend our sense of self as bounded. STEs are characterized by ego-attenuation, a reduction in self-other distinctions accompanied by a sense of oneness, a deeper sense of gratitude, a broader perspective of what it means to be human, and a prosocial desire to extend compassion and understanding to others (Piff et al. 2015, Yang et al. 2018). A variety of spiritual and therapeutic practices, as well as psychedelic drugs, have been shown to facilitate STEs and ego-attenuation (Letheby and Gerrans 2017, Bethelmy and Corraliza 2019).

Virtual reality (VR) offers new opportunities in this context. Glowacki (2024) outlined a novel aesthetic for designing VR experiences termed “numadelic” (derived from the Greek roots for “spirit-manifesting”). The numadelic aesthetic stands in contrast to the approach adopted by the vast majority of VR designers, which tends to emphasize photorealistic fidelity to content that resembles day-to-day phenomenology. In the numadelic VR experience described by Glowacki et al. (2022), individuals were represented as dynamically pulsating light energy with diffuse spatial boundaries, rather than as material objects with hard boundaries. Results revealed comparable ego-dissolving effects for psychedelic experiences and the numadelic VR experience. On self-report scales that measured mystical experience, ego-dissolution, and a communal sense of self, the numadelic VR participants scored similarly to people on medium-dose psychedelic drugs (Schmidt and Berkemeyer 2018). These findings suggest that numadelic VR can be used to deliver experiences beyond the constraints of normal waking reality that transcend day-to-day phenomenology (Riva et al. 2019).

Dreams are like VR experiences in some ways, but they can be distinctly hyper-realistic. Also, whereas a VR experience can guide cognitive processing within a controlled environment (Wilson and Soranzo 2015), dreams are not designed with a predetermined plan, the way a VR designer produces a VR experience. Nevertheless, can such crafted experiences also pervade our dreams through what can be called *dream curation*?

Dreaming has the potential to naturally lead to a STE; dreams are capable of producing profound changes in self-related conceptualization. For example, Hobson et al. (2014) proposed that within dreams, the brain updates world models and refines its ability to predict the causes of our waking sensations. REM sleep and dreaming have also been linked to memory consolidation, schema building, and emotional processing (Diekelmann and Born 2010, Wamsley and Stickgold 2019, Zadra and Stickgold 2021). Dream content often reflects our daily experiences, contributing to the consolidation of memories and the processing of emotions (Schredl and Hofmann 2003, Payne and Nadel 2004, Walker and Stickgold 2010). Dream experiences are often interwoven with emotionally significant stimuli associated with waking moments (Hartmann 2010, Solomonova and Carr 2019). Fragments of our daily experiences find their way into our dreams, such that dreams can influence how we navigate our waking lives (Cartwright 2010). Is there a reliable way to curate which themes from our waking moments become part of our dreams? Given that a waking numadelic VR experience can produce ego-dissolving effects (Glowacki et al. 2022), we sought to use these designed experiences to also influence dreams and further reshape waking perception.

One promising ingredient for reaching such goals is lucid dreaming. In lucid dreams, dreamers become aware that they are dreaming. While remaining in a lucid state, lucid dreamers can often take steps to influence the dream’s content, such as flying, modifying the environment, or making specific elements appear or disappear (LaBerge et al. 1981, Laberge and Rheingold 1990, Schädlich et al. 2017, Oudiette et al. 2018, Baird et al. 2019, Zerr et al. 2024). Lucid dreamers occasionally report transpersonal lucid dreams, defined by an expanded sense of self, in which they explore spiritual or religious themes in their dreams (Stumbrys 2018). Recent scientific investigations have also begun to characterize *Tibetan dream yoga*, a series of practices aimed at cultivating lucidity, psychological flexibility, imagination, and somatic awareness during dreams (Sheehy 2023, 2025, Torres-Platas et al. 2024). Researchers aim to characterize these practices and their benefits by recruiting experienced practitioners and using a multidisciplinary approach that combines overnight electroencephalography (EEG) recordings, micro-phenomenological reports, and humanistic methods.

One limitation of typical dreaming studies is that they rely upon participants’ post-sleep self-reports. Reported dream experiences may be biased by waking reflections, or in the extreme, they may not have actually occurred during sleep. Fortunately, studies using polysomnographic recordings can allow for the verification of lucid dreaming and of specific dream activities in real-time (LaBerge et al. 1981).

While sleepers are generally disconnected from the external environment, stimuli such as sounds, flashing lights, vibration, and odors can influence dream content and increase the likelihood of lucidity (Carr et al. 2020). Combining cognitive techniques, sleep disruptions, and external stimuli can also increase the likelihood of lucid dreams (Stumbrys et al. 2012, Erlacher and Stumbrys 2020, Erlacher et al. 2022, Carr et al. 2023). The use of these techniques in a laboratory has created new opportunities for two-way communication between the dreamer and the researcher (Konkoly et al. 2021). Prior studies have explored the extent to which lucidity can be enhanced by exposing participants to virtual environments with dream-like properties that differ from everyday waking moments (Gott et al. 2021). A VR flying experience in which participants navigated through floating rings induced flying dreams (Picard-Deland et al. 2020). Particularly relevant for the present approach, VR environments can enhance self-reports of awe (Chirico et al. 2017), which is a transcendental emotion linked to compassion and gratitude.

**Figure 1:**
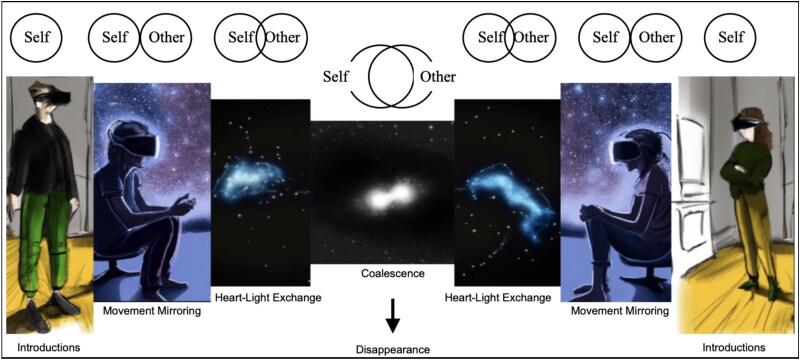
Overview of key moments during *Ripple*. The participants and facilitator started in separate rooms with their virtual headsets, entered the virtual environment as glowing purple and gold-colored energetic bodies, and introduced themselves. They faced each other, mirrored arm movements, and intentionally followed the pace of the other until they were moving in synchrony. Next, they exchanged the heart lights of their energetic bodies with each other. They merged their virtual energetic bodies into a single coalesced energy body (see Glowacki 2024 for details), blurring physical boundaries and inducing a sense of oneness. Then, participants sat down and breathed as their energetic bodies disappeared. The overlapping circles above each phase demonstrate an example of how *Ripple* can facilitate the merging of self and other. Self-other circles are inspired by the Inclusion of Other in the Self (IOS) scale (Aron et al. 1992). Images generated using DALL·E 3 by OpenAI.

Building on the results of Glowacki et al. (2022), this study aimed to investigate the combination of an ego-transcendent VR experience and subsequent lucid dreaming about that experience. We used a variant of the numadelic VR experience described above, known as *Ripple,* as illustrated in Fig. 1. We monitored polysomnographic data continuously so that during REM sleep we could present subtle auditory cues corresponding to moments from *Ripple* that participants selected as being most profound. We used signals from dreamers to timestamp when participants’ lucid-dream experiences occurred. We also collected qualitative data through surveys and interviews as an initial foray into whether lucid dreaming about *Ripple* could enhance its ability to promote compassion and ego-attenuation. Lucid dreaming in one participant was explored in greater detail by conducting micro-phenomenological interviews (Petitmengin 2006, Demšar and Windt 2024). We hypothesized that waking experiences of *Ripple* combined with auditory cues during REM sleep could result in *Ripple*-related experiences during dreams*.*

## Materials and methods

### Participants

Four healthy individuals who were frequent lucid dreamers ($\bar{\rm x}$ = 34 years old, range 21–43) were recruited via word-of-mouth. Three identified as female and one as non-binary. One participant identified as White, one as White and Asian, one as Black, and one as Latina. They each reported at least one lucid dream per month, with a mean of 3.8 lucid dreams per month. Data were collected from a pilot participant in which lucid dreaming about the VR experience was also noted, but those data are not included here as the same procedure was not followed. Participants were compensated $150 at the completion of the study and all procedures were approved by the local Institutional Review Board.

### Software and hardware

Participants used a Meta Quest 2 headset and hand controllers during *Ripple. Ripple* is a group VR experience, typically for two to four participants, although a maximum of eight is possible. *Ripple* and a tutorial on its use can be freely accessed through the Meta Quest app by searching “aNUmaXr.” Facilitated group experiences require an active server, which can be set up by contacting aNUma through their website (https://aNUma.com). *Ripple* uses an online cloud computing server to enable multiple participants to interact in the same virtual environment. Each participant has a virtual energy body with either a gold or purple color visible to other participants within the virtual environment, and virtual energy body movements correspond closely to each participant’s headset and hand controller movements. This setup allows for synchronous and shared interaction within the virtual environment. *Ripple* integrates new mechanics compared to prior numadelic VR experiences, including: (i) participants can spatially control the position of the light which corresponds to their heart light so that it merges with others when they move their hand controllers apart; and (ii) participants appear as energetic essences with dynamically changing spatial boundaries.

Prior to the start of the experiment, researchers were trained to facilitate *Ripple* by an expert facilitator (J.W.) over three sessions. The training focused on explaining the background of meditative exercises associated with the *Ripple* experience and presenting a framework to create a comfortable and reflective environment for participants.

### VR experience

Both *Ripple* sessions consisted of three phases. On average, Phase 1 lasted 10 min, Phase 2 lasted 30 min, and Phase 3 lasted 10 min. Participants received guidance on how to use the Meta Quest 2 device in advance.

#### Phase 1

Participants entered a virtual room where they engaged in the *Ripple* experience. To acclimate to the environment, participants were prompted to explore the visual space and experience their energetic bodies, with a central heart light, for about 2 min before a trained facilitator began with introductions. Participants had the ability to communicate with each other and the facilitator through a microphone and speakers in the headset. Participants introduced themselves, were asked to reflect on how they felt, and were reassured that anything they expressed within the virtual experience would be confidential (communication within the virtual experience was not recorded or analyzed).

#### Phase 2

Participants engaged in a series of group activities that used a pre-recorded guide inspired by Buddhist contemplative meditation exercises. These partnered practices included mirroring arm movements, exchanging light hearts with each other, disappearance into darkness, and the integration of virtual bodies into one energetic coalescence. Each activity was accompanied by its own distinct musical track.

#### Phase 3

The facilitator encouraged participants to virtually hold on to a moving energetic thread and reflect on their experience with the group for about 5–10 min. To guide the sharing, the facilitator asked questions including “how do you feel?,” “how does your body feel?,” and “which parts of this experience resonated with you the most?”

### Experimental procedure

**Figure 2:**
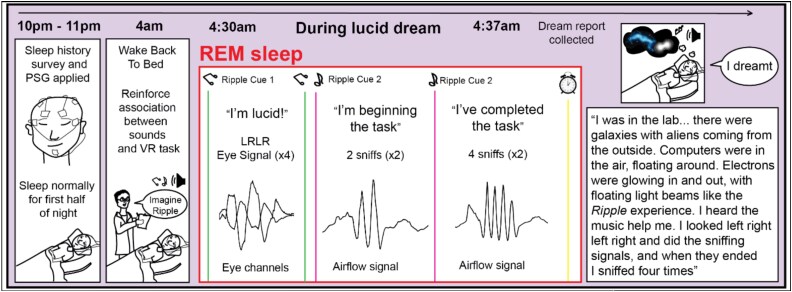
Example of the overnight protocol, timeline, lucid-dream signals, and dream report of a representative participant (participant 3) who had a lucid dream in the lab. The participant completed surveys and then slept normally for the first half of the night from 10 p.m. to 4 a.m., then she was awakened at 4 a.m. and listened to music from *Ripple* for 15 min, setting the intention to have a lucid dream and continue exploring *Ripple* in her dream. After falling back asleep, she entered REM sleep (central square), and music from *Ripple* was played softly to remind her of the task (cue 1 on the left). She signaled LRLR with her eyes once she became lucid, sniffed in-out twice when she began the task, and in-out four times after completing the task. The participant responded to 4 out of the 12 cues presented. She was then awakened and reported dreaming of *Ripple* and completing the signals.

The experiment incorporated VR and an overnight sleep session as follows. The first session in the lab included only the *Ripple* VR experience, providing participants with the opportunity to acclimate to the VR environment and reduce any potential novelty effects (Miguel-Alonso et al. 2024). Participants then went home and slept as usual for one week, and each morning they completed a short dream report survey and followed a written prompt guiding them in a 5-min exercise to reflect on *Ripple* and to notice any thoughts or feelings arising. This exercise was intended to help participants mentally prepare for their second session and set the intention to have a lucid dream about *Ripple*. Participants returned to the lab ~1 week later, 3 hours before their normal bedtime, and participated in *Ripple* for a second time. The overnight procedure during this second lab visit is illustrated for one representative participant in Fig. 2. After both VR experiences, participants completed a survey in which they were asked to describe the experience in their own words, identify a specific moment from *Ripple* which resonated with them the most, rate their sense of awe, and rate their experience of self in relation to other participants (Hanley et al. 2018, Kettner et al. 2021). Participants were asked to describe why they selected that moment and how they would like to continue exploring it in a lucid dream.

Next, participants were prepared for polysomnography (PSG) using a NeuroScan SynAmps system with a 1000-Hz sampling rate, including EEG channels F3, F4, C3, C4, O1, O2, chin electromyography (EMG), two horizontal and one vertical electrooculography (EOG) electrodes, two electrocardiogram (ECG) electrodes, and a nasal cannula to measure airflow. Volume intensities for music from *Ripple* were titrated before sleep to determine a minimally audible level as well as a comfortable level for sleep for each participant. These levels were determined by playing each sound very quietly from a speaker located on the ceiling of the bedroom and increasing the volume until participants could report hearing the sound (minimal level) and further increasing it until participants reported what would be a comfortable level for sleep.

Participants were told that they would be awakened after 5 hours of normal sleep (~4 a.m.) and spend 15–30 min awake, often referred to as the wake-back-to-bed method to promote lucid dreaming (LaBerge et al. 1994). During this awake time, they were encouraged to set an intention to have a lucid dream to continue exploring *Ripple* in their dreams, specifically the moment they found most profound. They were offered drawing paper, books on lucid dreaming, or videos about lucid dreaming activities to help with setting their intention. Most participants chose not to use the materials provided, other than the drawing paper. During the application of electrodes and for the remainder of the wake-back-to-bed period, music from *Ripple* was played in the room and participants were told to associate the music with their intention to become lucid and explore *Ripple* in their dreams. Participants selected a musical cue from the *Ripple* soundtrack corresponding to their preferred moment from *Ripple* (the options were: An Arc of Doves by Brian Eno, Safe by Julianna Barwick, and 1/2 Singing Bowl Ascension by Jon Hopkins). They were also asked to express how their musical preference connected to their experience of *Ripple*.

When participants entered stable REM sleep, they were cued with their selected music choice. Cues were presented at each participant’s minimal audible level and gradually increased to their preferred intensity (as determined before sleep). Sleeping position was not monitored, so perceived volume intensity may have varied throughout the night. Cues were stopped if any signs of arousal were detected (e.g. increased alpha level, increased muscle tone, movement). *Ripple* auditory cues were between 1 and 3 min in duration and were most often presented for their full duration. Experimenters stopped and restarted the music occasionally to counteract habituation and increase the possibility of participants recognizing the beginning of the music as a signal to become lucid. Dream reports were collected at the end of each REM period or ~1 min after the final lucid-dreaming signal.

### Lucid-dream task

Participants were instructed to signal with left–right–left–right (LRLR) eye movements upon becoming lucid in their dream (see lucid signal in Fig. 2). Next, we explained to participants that they could revisit their experiences from *Ripple* in a dream by re-entering the *Ripple* environment or reflecting on their most significant moment. We henceforth refer to these possibilities of re-engaging with *Ripple* themes as revisiting *Ripple*. Participants were also instructed to signal their progress in real time with sniffing signals. They were instructed to make two in-out sniffs as the signal for beginning to revisit *Ripple* and four in-out sniffs as the signal for completing revisiting *Ripple*. We chose these signals based on previous findings (LaBerge and Dement 1982, Mironov et al. 2018, Konkoly et al. 2024) that alternating large-amplitude sniffs can be distinguished from normal respiratory patterns.

### Sleep EEG

Data from scalp electrodes were filtered in MATLAB using a 0.3 to 35 Hz bandpass. EMG signals were filtered from 10 to 100 Hz. During periods when sweat artifact was present in REM sleep, scalp and eye channels were high-pass filtered above 2 Hz. Sleep data were scored by an experienced researcher (D.M.) according to standard criteria (American Academy of Sleep Medicine 2023). Eye movements and respiration data were reviewed from all nights. Potential lucid dreaming LRLR eye movements and sniffing signals were identified by D.M. and reviewed independently by a second rater (S.G.T.-P.), who was blind to the initial ratings. Scoring of respiratory and oculomotor signals by S.G.T.-P. was performed independently of dream reports. No lucid eye signals or multi-sniff responses were observed outside of REM sleep. Only signals upon which both raters agreed were included in Table 1.

**Table 1:** Overnight sleep data highlighting the frequency of lucid dreams and responses to auditory cues during REM sleep.

Participant ID	Dream report with elements of *Ripple*	Signal-verified lucid dream	Cues presented during REM	Signals completed during REM	Signals in response to cues	Duration of lucid dream (s)	Total Sleep Time (min)	Time in REM (min)
1	Yes	Yes	3	3	1	32	261	20
2	Yes	No	10	0	0	0	353	62
3	Yes	Yes	26	9	5	280	454	57
4	Yes	Yes	2	5	2	66	373	34

### Dream reports

When participants were awakened from sleep, they were asked the following series of questions.

(i) Can you tell me everything you can remember?
(ii) Did any of your experiences during sleep relate to the sounds or task?
(iii) Do you remember anything else?
(iv) Please try to recount in as much detail as possible the sounds you heard and signals you completed.

These reports were audio-recorded and later transcribed. Reports were then coded by two independent raters using an evaluation grid for elements of interest such as lucidity, the laboratory environment, signals, volitional influence over the dream, and false awakenings. Dream content was also analyzed for the presence of sensory elements related to *Ripple*. For example, reported dream content was coded for the presence of an energetic body or similar visual percepts, such as clouds or auras of light akin to those seen in *Ripple*. Other elements of *Ripple*, both visual, auditory, and kinesthetic (i.e. arm movements like those practiced during *Ripple*) were also noted. After completing their ratings independently, the raters met to resolve any discrepancies through discussion until consensus was reached. At-home dream reports collected via an online survey were coded using the same evaluation grid by the two independent raters. Completed evaluation grids for at-home dreams are available in the supplementary materials.

### Surveys

Survey data were scored for the presence of emotional experiences, which participants were asked to describe in a free-response format, shown in Table 2. Participants also completed an adapted 10-item “communitas” scale designed to measure participants’ feelings of interconnectedness (Turner 2017, Kettner et al. 2021), rated on a scale from 1 to 7 (strongly disagree to strongly agree). We chose this scale based on Glowacki et al.’s (2022) finding that communitas is increased after numadelic VR experiences, in which participants report intense feelings of togetherness and shared humanity, temporarily transcending social structures.

**Table 2:** Excerpts from survey responses to the question, “Please describe *Ripple* in your own words, including any thoughts, memories, sensations, emotions, or feelings that arose for you throughout the experience.”

Participant	Reported experience after *Ripple* 1	Reported experience after *Ripple* 2
1	“Smoky ethereal bodies. Synced hand movements. Breathing in tandem. Space that seemed limitless. More intimate.”	“My arm movements and breaths felt synchronized. It reminded me of a dream I once had where I died. I was a spirit and was looking to find another spirit.”
2	“Blending with other people. Resistance to give my heart to someone else.”	“Merging the bodies gets easier every time. Less reluctant to give everything I have and receive from others. I’m able to smell the blob of energy as a fresh sea breeze. I realized how easily I can trick my body.”
3	“Absolutely amazing. I felt like I was in space and a ball of energy. I feel I am a part of something great.”	“An awake out-of-body meditation experience. Allows me to connect with my inner child self. I really love how I interact with my partner.”
4	“Calming and soothing. Allowed for deep reflection and retrospection about one’s connection with the self and with others.”	“I was expecting what to do already. Still excited about going through everything and not having a body.”

### Micro-phenomenology

Participant 3 met with an interviewer (E.D.) who has extensive training in the micro-phenomenological interview method (Petitmengin 2006). This approach has been recommended for in-depth explorations of the phenomenological dynamics of dream experiences (Demšar and Windt 2024), complementing immediate post-awakening dream reports, which often focus on the narrative content of dream events and lack fine-grained phenomenological detail.

Two interviews were conducted over video calls after the laboratory session, one lasting 76 min and the other 119 min. These interviews enabled the participant to further explore her lucid dream episode and delineate its precise temporal progression. She provided a detailed description of the emergence and progression of elements of *Ripple*. Details from the micro-phenomenological investigation can be found in the supplementary material and include: (i) representative excerpts to summarize the participant’s experience; and (ii) the participant’s drawing of the experienced dream scene.

### Final interviews

One week after their final lab visit, participants were invited for a 30-min semi-structured zoom interview conducted by D.B.E. Participants were encouraged to reflect on both their waking and dreaming experiences of *Ripple*, describe their emotions, and report any consequential dreams during or after the study. This interview provided participants with the opportunity to reflect on their overall impressions and provide qualitative reports on any broader impacts of the study. The following questions were included.

(i) Did your *Ripple* experience change between your first and second session?
(ii) Was there a part of participation in the study that resonated with you the most?
(iii) Did you experience any unexpected emotions during *Ripple*?
(iv) How did the overnight experience impact your perception of *Ripple*?
(v) Did you have any lucid dreams? If so, did they connect with any aspects of *Ripple*?

The interviewer also asked follow-up questions in response to participants’ answers. Extended sections of participants’ final interviews can be found in the supplementary materials.

## Results

**Figure 3:**
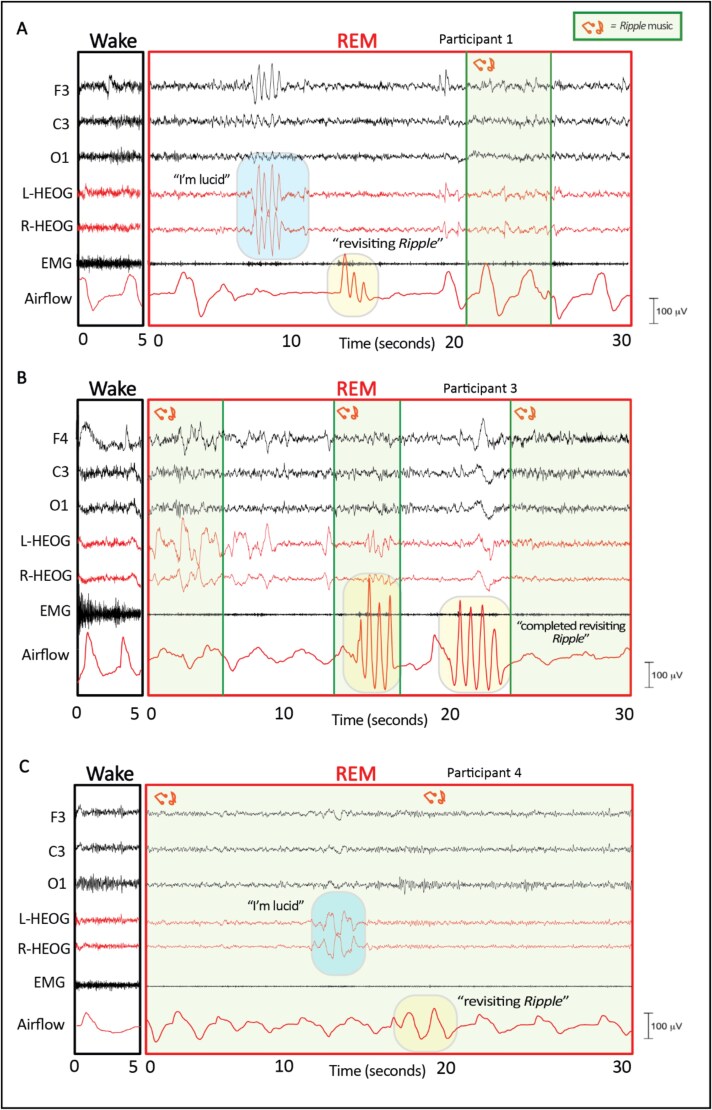
Examples of signals produced during REM sleep. Comparing sleep physiology during a 5-s period of wake (left) and a 30-s period of REM (right) demonstrates typical characteristics of REM sleep. LRLR eye signals indicated lucidity in the dream, two sniffs indicated beginning to revisit *Ripple*, and four sniffs indicated completion of revisiting *Ripple*. (A) Participant 1 completed the lucid signal, followed by three sniffs (potentially an attempt at completing two sniffs), 25 s later, she completed two sniffs again. (B) Participant 3 in REM sleep completed four sniffs twice to indicate that she had completed exploring *Ripple* in her dream. She first signaled becoming lucid in her dream 4 min and 40 s prior, and during this REM period, she responded to 4 out of 12 auditory cues with either LRLR or sniffing signals. (C) Participant 4 in REM sleep completed the lucid signal, followed by two sniffs. Music from *Ripple* was presented 30 s prior to her signal and played continuously throughout the episode. She completed four sniffs before awakening.

All participants recounted lab dreams containing elements apparently from *Ripple.* The duration, intensity, and qualitative aspects of these experiences, including sensory and thematic components, varied among participants. Participants’ descriptions of *Ripple* from the post-*Ripple* survey are shown in Table 2 and lab dream reports are shown in Table 3. Three of the four participants produced signal-verified lucid dreams by performing the predetermined LRLR eye signals during the overnight session in the lab. Examples of EEG data, auditory cues, and lucid dream signals are shown in Fig. 3. The frequency of lucid dream signals and related cues presented during REM sleep is shown in Table 1.

**Table 3:** Excerpts of lab dream reports with VR-related content.

Participant	Dream report
1	“My dream started and there were no visuals or anything. So then I did the lucid signal and then I thought, ‘Ok, I’m gonna start trying to do the conjuring, the *Ripple* effect.' So I did the signal and I was trying to think about that and have it appear but it was still really dark. And I was in the lab, so it was still dark and I couldn’t really get anything to appear. Then I got out of bed and I was moving around, trying to make something more happen, and trying to make the lucid dream get brighter. I thought ‘brighter, brighter’ … and during that time I was doing the arm movements. At some point I started to hear a bunch of radios, like TVs on, like a bunch of people talking. Then it sort of went away. I was saying in my head ‘louder, louder’ because I wanted more things to happen and there was nothing really going on in the dream except for being in this dark room and moving my hands in the *Ripple* shape. And then I started to go towards the door, but then I think I woke up.”
2	“I was at a grocery store but also kind of a hotel and we were staying inside. My friend was arriving and we were talking and laughing, so it was a friendly conversation. And I was like, ‘Yes, I’m going’, and then I heard the music and then I was like ‘Wait, wait, the music is like … Oh! I have to do something’. And that’s when I rushed to wake up. When I was in hypnagogic sleep I started seeing a lot of lights, that purple light that was in *Ripple*. And some lights were small spheres with many different colors inside in a drop shape. They were coming up with a lot of light, and the purple blob was coming and disappearing.”
3	“I was in the lab and there were hikers outside. We were getting ready because the aliens were coming from the outside, and on the inside it was like *Ripple*. It kind of reminded me of the movie ‘Batteries Not Included.’ They were glowing. There were computers in the air, floating around. And I was looking up and there were electrons glowing in and out. I kept turning on my side; I was turning back and forth, back and forth, like rocking from left to right. And then I heard the music help me. I remember doing signals, looking left to right left to right. And when they ended, I think I sniffed four times. I was looking forward to that and every time the music played, the jellyfish would glow in the air. And then I would rock back and forth on my side, up and down, but the door was open. I could see on the outside people working in the lab, and you would come in, open the door, and be talking to me. You were coming in talking to me, doing the electrode things.”
4	“Before I went to sleep I set the intention to ‘find peace’ because I just had a fight with my mom before coming to the lab. In my dream I was in the swamp collecting sticks and I slipped and that’s when I realized I was in the dream, so I did the eye signal. Then I flew out of it, flying up into space, and it was really pretty. That’s when I did the two breaths. My classmate was there and she turned into my mom. We held hands and enjoyed being together. When it was done I gave four breaths. … I opened the door and realized I was dreaming so I did the eye signals. I was in a forest but there was an art supply area right outside. And then my former castmate was there and she gave me life advice on how to be spontaneous and connect with other people not because you want a relationship but because that’s just how life is. I did the two sniffs when she appeared and four sniffs at the end.”

In follow-up interviews, participants reported that after their overnight *Ripple* session, they had enhanced feelings of interconnectedness and compassion compared to prior to the beginning of the study. For example, participant 4 described their favorite part of *Ripple*, “I liked when our bodies changed into the same color… we were doing the same thing, joining together, and being ‘one’, becoming the same thing. It was interesting, especially since we already didn’t have physical bodies. The things that represent our physical bodies were reflections of each other. Almost like an erasure of differences or divisions.”

Participants 2 and 3 reported experiences of heightened waking sensory perception both during *Ripple* and for several days after their *Ripple* experience. For example, participant 2 described, “… my brain started giving me sensations… the experience of *Ripple* was fooling my brain. The sensations and even (imaginary) smells. At some point, I was even experiencing smells. I was feeling it in my body. I was very immersed. I was like, ‘this is crazy! This is not happening!’ but my brain already reconstructed the smell, reconstructed the feeling when we were merging in *Ripple*. It’s like I could feel it in my skin. It was like how the [energetic bodies] were coming together. I was like, ‘This is just insane’. And then that carried over into my week where I was like, this is so powerful on how you can fool your brain to believe the things that happened.”

Several participants reported that their lab lucid dreams included close family members and themes of collaboration. For example, participant 4 described their lucid dream, “I was in a car, flying up into space with my friend, but my friend turned into my mom. And we held hands and reconciled because earlier before the study, I had a fight with my mom.” Participants also questioned their identity, with questions such as “Who am I?” after the first *Ripple* experience, particularly after the exchanging of their energetic heart lights. For example, participant 2 reported, “I remember being very shocked when the experience said to give everything you have, such as your best qualities, to the other person, everything. Then I remember feeling, okay. I’m going to give everything. But what am I going to stay with? I felt resistance about giving. Right? I was like okay... then who am I?”

Analysis of the two micro-phenomenological interviews with participant 3 revealed six phases of her dream: (i) a nonlucid phase in which the dreamer is lying in the lab bed, peacefully observing the room while feeling awake and aware of currently taking part in the experiment; (ii) a non-lucid phase in which she starts noticing inconsistencies in the dream experience but explains them away; (iii) a pre-lucid phase in which she starts explicitly wondering about these oddities, realizes she is dreaming, and performs the eye-movement signal; (iv) a lucid phase in which she is expecting an experience of *Ripple* to happen and is waiting for it to begin; (v) the *Ripple* experience itself (also a lucid phase), objectively marked by the two- and four-sniff signals at the start and the end; and (vi) a final lucid phase after the end of the *Ripple* experience during which she is hearing music, the scene darkens, and she eventually wakes up (Phase 6). Detailed summaries of all phases with representative interview excerpts are available in the supplementary materials.

In Phase 4, the dreamer was preparing for the *Ripple* experience: she knew that it would happen as part of the experiment, and that she just needed to “allow” it:

Something told me that I need to just look and allow it to happen … by being still. … It got recorded in my head that it was supposed to happen. … [Daniel] didn’t say … IF you see it, he said WHEN you see it, when it starts, this is what you do. … It was already put into motion that it was gonna happen. … I prepared myself to look around and observe when it was gonna happen.

Phase 5 began when the dreamer noticed two luminous clouds of purple and orange gas above and to her left. The clouds were illuminating and dimming rhythmically—simultaneously expanding and contracting—for four cycles in total before dimming out; this was experienced as lasting around 2 min.

It was in that gassy cloud. … It looked just like me and Blaise when I was in the virtual reality thing. … I just watched. I allowed it to do like this [pulsing gesture], the clouds to go light, to get dimmed, and go in and out. … Illuminating in and out. … It’s just like they were breathing … They sent the signal of what I experienced in the VR … That’s the only way I knew that that was the experience [i.e. the ripple experience].

During this experience her attention was locked on the clouds, and it felt like she’s “looking for answers” related to her childhood by focusing on the light:

My focus, I had tunnel vision, straight on the clouds. … I didn’t want to look at nothing else. … That’s why I felt special: I get to see this. … It was a calmness … [an] exciting calmness.

I was just looking up at it … and it felt so good, just looking at it. It’s like a silent communication. … It’s something in me that knows: this is what I’m supposed to be doing. This is what I’m supposed to be looking at. … It just felt like: that was how I was gonna get my answers.

The experience was strongly positive, with feelings of awe, connection, and belonging: “I felt accepted. … I felt I belong. … I felt, I feel beauty.” There was a strong sense of meaning linked to the prior VR experience: it felt as if the clouds were communicating with the dreamer and beaming back the energy she had projected in the VR experience:

It was just like what I did in the VR exercise: When you breathe in, it’s tight. When you breathe out, that’s when they beam open. So, they [the clouds] were blowing towards me, so I could receive the energy. … That’s what feels like the connection. … A mirror image of what I was doing in the VR. … I was giving it that same energy, so the dream was giving it to me back. … [In the VR] I was the purple cloud, but now I could see the clouds.

It felt like warmth … located on my face, up here [showing]. Like a sunlight hitting, beaming on you. … It’s hard to explain. … I’m observing, but I’m also taken in. … If you were to take your hand and put it next to your face, you could feel the warmth on your hands ... I felt like it was a presence there.

Her sense of embodiment also shifted: she no longer felt fully inside her body (as she had in the earlier phases), but felt more like a “floating head,” and she noted a decrease in the sense of boundary and separateness:

I don’t even really feel like I was in my body. I feel like I was a part of that, it was like a mirror … I felt like I was like a part of that experience of what it is I was seeing. …I felt airy … like things could come through you, like you’re floating, like you’re lighter, I felt light. I felt connected. I felt like I could float and move... I felt soft.

**Figure 4:**
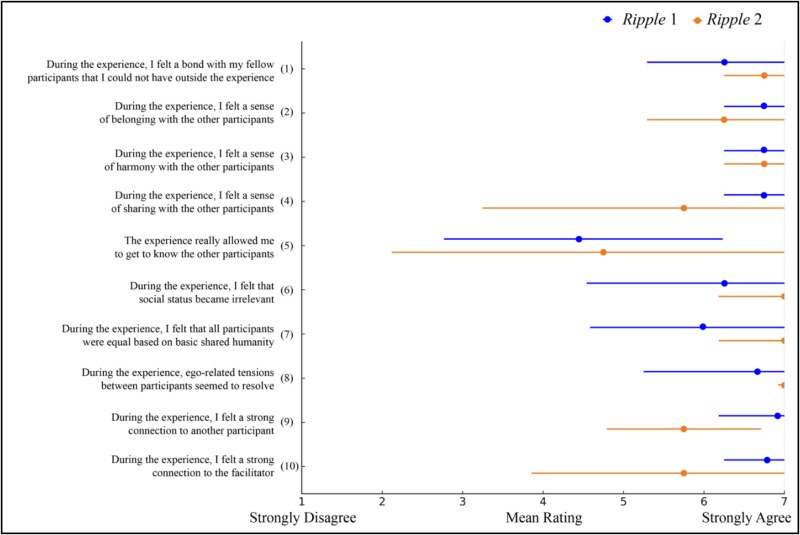
Survey responses to the Communitas scale were similar after both *Ripple* experiences. Items 1–8 assess participants’ sense of community, item 9 assesses participant-to-participant connection, and item 10 assesses participant-to-facilitator connection, with ± 1 SD error bars.

Post-*Ripple* Communitas survey scores, shown in Fig. 4, were high after both *Ripple* experiences (first session M = 44, second session M = 43.75 out of 56). At-home dream reports contained lower rates of *Ripple*-related dreaming and lucid dreaming compared to lab dream reports, as shown in Table 4. Participants completed a total of 63 at-home morning surveys, which included 52 (82%) mornings with recall. In 8 (15%) of these dreams, participants reported some aspects of the dream relating to *Ripple*. Participants reported lucid dreams in 14 (27%) of their at-home dreams. Participant 1 did not regularly complete the morning surveys (only two responses). Participants 2, 3, and 4 completed the surveys more regularly, averaging 20.3 morning survey responses. Overall, at-home dream reports contained fewer reports of lucid dreaming or *Ripple*-related dreams compared to lab dreams. At-home dream report data are available in the supplementary materials.

**Table 4:** At-home dream reports had lower rates of lucid dreaming and *Ripple*-related dreams compared to lab dreams.

Participant ID	At-home morning survey responses	Responses with a dream report	Lucid dreams	Dream reports with *Ripple*-related elements
1	2	2	0	0
2	25	23	4	2
3	14	7	3	4
4	22	20	7	2

## Discussion

This study demonstrated that dreams about *Ripple* were produced in four frequent lucid dreamers. Supportive evidence included dream reports obtained during awakenings in the lab and electrophysiological signals of lucid dreaming and *Ripple* experiences made in the context of REM periods verified with polysomnography. This combination of evidence strongly suggests that the experimental procedures facilitated lucid dreams pertaining to the prior VR experiences. Our previous research using auditory cues during sleep (e.g. Konkoly et al. 2021, Paller et al. 2021, Carr et al. 2023, Konkoly et al. 2025) supports the notion that cues in the present study helped produce both lucidity and the intended dream content. By facilitating deeper engagement with *Ripple* through lucid dreaming, this study also explored the potential of lucid dreaming to enhance and extend the impact of VR on compassion and ego-attenuation. Qualitative analyses revealed that these dreams not only incorporated sensory elements from *Ripple*, but also featured themes of compassion and unity, suggesting an integration of VR-induced phenomenological states into dream content.

Beyond the waking VR experience, the additional opportunity to explore *Ripple* within a dream—a primordial VR with all the senses operative and only subtle deviations from waking experiences—provides the potential for revisiting *Ripple* in novel ways, perhaps in quite expansive ways, potentially leading to transformative effects. Whereas inhabiting an energetic body during *Ripple* can be a profound experience, it may be colored by cognizance of the fundamentally artificial constitution of VR.

A dream, on the other hand, entails an experience of complete immersion that feels very real—so real that dreams are almost always taken as waking reality at the time they are experienced. Lucid dreams are rare precisely because dreams characteristically seem so real. Consider the dream of participant 2, where she experienced going to a hotel and talking with friends but did not realize she was dreaming. Lucid dreams offer the profound opportunity to recognize the constructed nature of one’s experience and gain volitional control over dream content. Speculatively, lucid dreamers may be influenced by knowledge that is generally not accessible during wakefulness. Exploring the far reaches of *Ripple* in a dream may allow participants to integrate impactful moments into their conceptions (or revised conceptions) about the world. Our study builds upon previous research showing that lucid dreamers can modify their dream content and provides evidence that the combination of VR and lucid dreaming can be synergistic in producing STEs. Participants’ control over their dream environment in our study allowed them to create unique narratives and expand upon specific moments and themes from the VR experience, which is seldom possible in the waking state, even with VR. A story from participant 4 serves as a marker of the potential for personal breakthrough experiences; she had an argument with her mother the night before her overnight visit to the lab and then had a lucid dream in which she flew into a *Ripple*-like space to hold hands with her mother and experience the joy of being together.

Micro-phenomenological interviews allowed participant 3 to provide a rich description of her key dream experience. The step-by-step dynamics of the dream episode were clarified in ways not evident in the initial dream report, including the associative metaphors produced in her initial post-awakening report. These interviews highlighted the emergence of *Ripple* in the dream, revealing the attentional disposition with which the dreamer prepared for the experience and “allowed” it to happen, as well as the role of experimenter instructions. Despite some ambiguity (e.g. interpreting divergence between the interview and the initial report), the findings illustrate the potential for micro-phenomenological explorations to help link the nuances of dream experiences to behavioral and physiological markers.

Final interviews helped to reveal specific themes that emerged after the combination of *Ripple* and dreaming experiences. Participant 4 reported a profound experience of interconnectedness and ego-dissolution. Participants 2 and 3 reported heightened waking sensory perception, such as touch and smell, for several days. Participant 4 entertained themes of reconsolidation and collaboration based on lucid dreaming in the lab.

Communitas survey scores were high after both *Ripple* sessions (Fig. 4), echoing findings from prior research on numadelic VR experiences (Glowacki et al. 2022). The numadelic aesthetic may be particularly well suited to inducing experiences of interconnectedness and community. No changes were observed between the first and second *Ripple* sessions, although the limited number of datapoints in this study warrants caution in interpreting the generalizability of these results.

Dream reports tended to include *Ripple* environmental elements rather than plot elements (Table 3), which may be because *Ripple* is an immersive, aesthetic experience, not an agency-focused experience. Additionally, participants’ reports of awe during *Ripple* may play a role; awe has been shown to influence how individuals relate to and integrate their sense of self with their environment and other people (Keltner and Haidt 2003, Pizarro et al. 2021). In contrast, lucid dreams are typically characterized by self-directed experiences (Kahan and LaBerge 1994). When participants attempted to continue *Ripple* in their dreams, the awe-inspiring nature of *Ripple* may have contributed to the prevalence of environmental elements over narrative-driven content. A question for future investigation is thus whether transcendent VR can reliably shift the fundamental nature of dream experiences in these ways.

Amidst the insights afforded by this study, several limitations must be considered. Notably, our sample included only four frequent lucid dreamers, all living in the Chicago area. Future studies are needed to probe the generalizability of these findings across different populations. Additionally, the absence of a control group restricts our ability to solely attribute observed changes to the dream intervention, as opposed to external factors such as time or repeated exposure to *Ripple*. The absence of post-overnight survey measures limits our ability to compare participants’ responses before and after lucid dreams. Future studies could explore the effects of repeated exposures to *Ripple* and participants’ reports of interconnectedness. Given the multisensory nature of the VR experience, it may also be valuable to explore using multimodal cues during sleep, which have been relatively unexplored compared to auditory cueing paradigms (Salvesen et al. 2024). Given recent demonstrations of the potential for sleep reactivation of waking experiences to aid in the treatment of nightmares or PTSD (Schwartz et al. 2022, van der Heijden et al. 2024), such approaches could perhaps benefit from additional VR components.

The injection of VR into dreaming could also provide innovative insights at the intersection of waking, dreaming, and personal transformation. VR’s unique advantage of tailored environments can be utilized with a wide variety of valuable goals. Our findings underscore a way to expand VR’s benefits via VR-based dreaming. This study provides preliminary evidence from an admittedly small number of participants, but the proof-of-concept demonstration opens the door for future research to now test the degree to which lucid dreaming combined with VR can benefit psychological well-being. In particular, we envision many ways for dream content to synchronize with ego-attenuation and the perpetuation of awe in VR environments. By bridging the realms of virtual waking and dreaming states, this study opens new avenues for understanding how combining immersive technologies and sleep-engineering technologies might be leveraged for therapeutic and personal growth in waking life.

## Supplementary Material

NCONCS_Supplemental_Revised_niaf017

## Data Availability

Study materials and data are publicly available at: https://osf.io/sdh7e/.
