# Supplementary material for: Lucid dreaming of a prior virtual-reality experience with ego-transcendent qualities: a proof-of-concept study
Source: Neurosci Conscious. 2025 Aug 5;2025(1):niaf017. doi: 10.1093/nc/niaf017 (PMC12342170; doi:10.1093/nc/niaf017)
Supplement: NCONCS_Supplemental_Revised_niaf017 [file nconcs_supplemental_revised_niaf017.pdf]

Lucid dreaming of a prior virtual-reality experience with ego-transcendent qualities: A proof-of-concept study

SUPPLEMENTAL MATERIAL

This supplementary material includes:

1. ***Ripple*** **post-experience questionnaire**
2. ***Ripple*** **revisiting prompt**
3. **At-home morning survey**
4. **At-home dream report responses**
5. **At-home dream report coded evaluation grid**
6. **Lab dreams coded evaluation grid**
7. **Final debriefing interview questions**
8. **Participant 3 drawing of dream**
9. **Participant 3 micro-phenomenology interview excerpts**

Ripple post-experience questionnaire

Northwestern QUALTRICS

What is your participant ID number?

[ ]

What is today's date:

[ ]

Who was the host for your experience?

- ○ Blaise
- ○ Daniel

What is your age in years?

[ ]

What is your gender?

- ○ Male
- ○ Female
- ○ Non-binary / third gender
- ○ Prefer not to say

How many times have you had a virtual reality experience in the last 12 months? (not including any VR experiences for this study)

[ ]

Have you taken part in a "Ripple" or "Isness" VR experience before?

- ○ yes
- ○ no

Please describe the "Ripple" experience in your own words, including any thoughts, memories, sensations, emotions, or feelings that arose for you throughout the experience.

Think back on the "Ripple" experience and identify a specific moment that resonated with you the most. It could be positive, negative, or neutral.

How much did the "Ripple" experience bring about the feeling of awe for you? Furthermore, how much was the experience beautiful for you (however you define beauty)?

How strong was your feeling of awe?

- ○ 1 - Not at all
- ○ 2 - Weak
- ○ 3 - Medium
- ○ 4 - Strong
- ○ 5 - Very strong

Did you have any of these bodily effects during the experience? Tick all that apply.

- ☐ Body aches
- ☐ Headache
- ☐ Sweats or chills
- ☐ Trembling
- ☐ Crying
- ☐ Other [ ]

Please indicate how strongly you agree or disagree with the following statements about your Isness ceremony experience, taking into account that 1=Strongly disagree and 7= Strongly agree.

	1	2	3	4	5	6	7	n/a
During the experience, I felt a bond with my fellow participants that I could not have outside the experience	○	○	○	○	○	○	○	○
During the experience, I felt a sense of belonging with the other participants	○	○	○	○	○	○	○	○
During the experience, I felt a sense of harmony with the others	○	○	○	○	○	○	○	○
During the experience, I felt a sense of sharing with the other participants	○	○	○	○	○	○	○	○
The experience really allowed me to get to know the other participants	○	○	○	○	○	○	○	○
During the experience, I felt that social status became irrelevant	○	○	○	○	○	○	○	○
During the experience, I felt that all participants were equal based on basic shared humanity	○	○	○	○	○	○	○	○
During the experience, ego-related tensions between participants seemed to resolve	○	○	○	○	○	○	○	○
During the experience, I felt a strong connection to another participant	○	○	○	○	○	○	○	○
During the experience, I felt a strong connection the facilitator	○	○	○	○	○	○	○	○
During the experience, I felt a sense of connectedness with others based on our common humanity that I think will persist for a while	○	○	○	○	○	○	○	○
I anticipate that the impact of the experience will have a lasting effect	○	○	○	○	○	○	○	○

Please read each statement and indicate the extent to which you agree with each statement. In other words, how well does the statement describe what you just experienced, just now? Taking into account that 0=Not very much and 100=Very Much

	0	10	20	30	40	50	60	70	80	90	100
I experienced all things seeming to unify into a single whole											
I experienced all sense of self and identity dissolve away											
I felt surrounded and filled with a blissful warmth or energy											

Ripple Revisiting Prompt

The Ripple experience involved engaging in movements with a partner. At a particular moment, you and your partner stood an arm's length away from each other and mirrored each other's movements, raising and lowering your arms. As you performed this exercise, you synchronized your breath with that of your partner, inhaling as your arms rose and exhaling as your arms descended. Later, you practiced the Tonglen motion alongside your partner. As your arms extended outwards, you observed your heart light emerging from your energy body. This motion enabled the heart lights to shift back and forth between you and your partner, symbolizing self-exchange. During this exchange, you were prompted to reflect on the sensations associated with giving and receiving, exploring whether one aspect presented more difficulty than the other.

1. Close your eyes and pause for half a minute as you try to replicate the experience in your mind as strongly as possible. How did that gesture make you feel? Did it enhance your sense of connection or empathy towards your partner? Can you recall any specific thoughts or emotions that arose during this interaction?

At one point in the Ripple experience, you were instructed to turn away from the group and practice the Tonglen motion. As your heart light extended from your energy body, you were asked to find stillness after your exhale and observe the suspended heart light. After remaining still for a brief period of time, the heart light began to dissolve away. Then, during your inhalation, your heart light reappeared as you brought your arms back towards your heart.

2. Close your eyes and pause for half a minute as you try to replicate the experience in your mind as strongly as possible. Reflecting on the practice of holding your heart light out and watching it fade as you remained in stillness, how did this experience affect your overall sense of presence or mindfulness? Did it evoke any particular sensations or insights about yourself or your connection to your surroundings?

During the Ripple experience, there was a moment of complete energy coalescence with the group. The group came together in the center, allowing their energy bodies to overlap and merge. As you joined the others in this shared space, you were encouraged to move your arms around freely, unrestricted by any self-other boundaries, allowing your movements to flow naturally.

3. Close your eyes and pause for half a minute as you try to replicate the experience in your mind as strongly as possible. When you participated in the complete energy coalescence with the group, how did this interaction impact your perception of connectedness with others in the group? Did it evoke any specific emotions or sensations that stood out to you?

At-home dream report survey

Northwestern | QUALTRICS

Participant ID:

Do you remember any dreams from last night?

- ○ Yes
- ○ No

Describe everything you can remember from your dreams and/or other experiences during sleep, including any details you can remember such as the sequence of events, your thoughts, sensations, and/or feelings.

Do you see any ways in which your dream(s) incorporated aspects of the Ripple virtual reality experience that you participated in?

- ○ Yes
- ○ No

Describe how the experience was incorporated in the dream.

Were you at any point aware that you were dreaming while still asleep?

- ○ Yes
- ○ No
- ○ unsure

If you were lucid, please describe the moment you became lucid and your lucidity throughout the dream.

In your waking life over the last 24 hours, have you had any experiences that strongly resonated with your ripple experience? If so. please explain.

At-Home Morning Survey Responses

Participant	At-home report number	Do you remember any dreams from last night?	Dream report	Do you see any ways in which your dream(s) incorporated aspects of the *Ripple* virtual reality experience that you participated in?	Describe how the experience was incorporated in the dream.	Were you at any point aware that you were dreaming while still asleep?
1	1	Yes	I was on a class trip at a hotel. I had a new boyfriend. He was hitting on me in real life on the trip.	No		No
1	2	Yes	In real life I applied my discord app to receive discord privilege intents. I got rejected and was chatting with the person who rejected me via email. In my dream I continued the conversation.	No		No
2	1	Yes	It was very long and somehow the whole sequence of events was in a restaurants and involved food. I dreamt of my dead grandma as if she was hosting and I remember trying to order a mescal drink in one of the restaurants. Then we loved to another restaurant and I ordered carne asada.	Yes	I guess there was not exct incorporation but the experience made me realize that I was grasping very strongly to my identity. The feelings evoked during the experience kind of repeat in the dream like always questioning the self.	No
2	2	Yes	I was first in a meditation or some kind of spiritual regreso. We were sleeping there with a bunch of people, then the place of my retreat converted in my office and I was convincing a fiend to share the space with me. Then I went to explore the area and I was following a group of Emu birds and one of them attacked me.	No		No
2	3	Yes	We were traveling in like a camp and when the camp finished my best friend and I went to the bar of the camp and we found a lucid dream celebrity and we were going to try the conductance of the shampoo head and shoulders in the skin. We decided to do a live demonstration of the EEG in the bar.	No		No
2	4	Yes	It was a very very long dream, in brief, I was in a vacation with many people and there were a couple of celebrities in the group. I arrived to the house of a friend and I brought 2 women with me (I don't know who this people were) and in the house of my friend we had the view of a big river and there were species of animals that have been extinct. The water was so transparent that I could see the details of these massive animals with very strange forms.	No		No
2	5	Yes	The dream happened during a few days we were in some sort of resort and we were sleeping in communitarian rooms. I had to sleep at some point with unknown people and my mom was in a different room but they were all connected. We had vip passes for some meals in the restaurant that had a pool inside.	No		Yes
2	6	Yes	I was an orphan in some kind of a heaven and I knew that I had a special power and I needed to use it. Finally, I meet someone that was supposed to take care of me and he gelt ill. I was very sad but I the same time I knew I had a special mission.	No		Yes
2	7	Yes	It was a long dream and I remember a lot but some parts of it was a trip with a bunch of people in a tour to different places. There was something that happened to one of the persons of the group that was very depressed. A bunch of people were helping her to pass her time.	No		Yes
2	8	No				
2	9	Yes	A lot of the topic on my dreams was about travelling and being in an airport. .	No		No
2	10	Yes	I was planning to have some people over for dinner and I was organizing with someone else to make it current after a class we all were having. We organizing and there was a lot of panning involved. Then I had to cut my friends hair.	No		No
2	11	Yes	I was in a social gathering where I was hosting. I had family, friends and many unknown people. We were reunited around a tree.	No		
2	12	Yes	I was setting up a business and I was trying to figure out how to do it. We were in some kind of institution where I was working there were some lab test I was running.	No		
2	13	Yes	I was organizing a Christmas party with bumblebee the transformer. I wanted to make it different so I wanted to have ophiuridae petting but remembered I had a dream like this before and I was not sure I dared to touch them in my previous dream. I was with a bunch of people from my elementary school.	No		Yes
2	14	Yes	It was weird because the last part I memeber I was in two places at once. In one place I was witnessing someone that became tiny like the size of a thumb, to scape and find her Prince Charming. They were in a boat and she scaled in the hair of a black gin real life and they left the boat in the beach.	No		No
2	15	Yes	I was organizing an event and we were having over many friends from childhood, hosting families, etc..we were giving an evil cookie that curse us in some unknown way and we were working to get rid of the u known curse by just talking about it. .	No		No
2	16	Yes	I was in a social event and I don't remember much more than being sitting in a table with a bunch of people.	No		No
2	17	Yes	I was trying to go an Office Depot to ask for their big care the next day. I don't remember why I desperately needed that car. Before that I was with a group of people that I didn't know but there was a lot of pretending and showing off.	No		No
2	18	Yes	I have to submit an application so all I remember was related to that application. I was writing it and developing a unicorn visual method for this proposal. My dead grandfather appeared in the dream, he died of Alzheimer's and in the dream he was all lost.	No		No
2	19	Yes	This dream is weird to explain but each French of breathing had a particular color and it became a persona. So if I was breathing quickly the breathing was becoming green and there was a green character that appeared. Like that I created like many characters with many colors that were dancing around.	No		No
2	20	No				
2	21	Yes	I was bitten by a black a white type of air Medusa that I have never seen before. I remember being extremely scared when the black and white air Medusa bit me. I tried to hit it so it could let me go but it didn't.	No		No
2	22	Yes	I had very repetitive dreams a bit frustrations, I was trying to solve a problem and I couldn't and I was repeating it over and over.	No		No
2	23	Yes	I remember I was in a park that was kind of a maze. I wanted to get out quickly but it was designed to stay. I was with 3 boys that I found in the way with a Slavic names and I was taking care of them, they were lost and scared.	No		No
2	24	Yes	The first night of the study after the lab, I had a dream where I was telling someone I didn't understand why rape was so strong and traumatic for some people. Right after I started getting in the mind of people and I saw a man that had raped a young woman. I could see how she was feeling and feeling everything she was feeling.	Yes	I became part of the other peoples minds and feelings. I was able to feel the girls feeling and to know somewhat telepathically what has happened.	No
2	25	Yes	I was scraping from a fantastic knight that was fighting another one with their flying horses. I was walking through train tracks and I arrived to a place where they were making human dolls. .	No		No
3	1	Yes	I had a dream I was in a food court buffet. This dream was from my college years and I was going from food vender to vender eating all I want when I want. When the cook served me my meal, the plate/bowl was filled with colorful airy gasy/smoke.	Yes	The food served in the dream was made of colorful smoke/gas. This reminded me of the gasy/smoke experience of me and the other participant.	Unsure
3	2	No				
3	3	Yes	Last night I had a dream I was a burning Phoenix flying across an ocean at night. I felt so powerful and I roared so loud and when I landed I became a human. I notice I only became a Phoenix when I get ready to fly in the air.	Yes	Yes became the phoenix bird symbolizes immortality, resurrection and life after death. I felt at peace in this moment and I was so high in the sky so spiritual and the stars in the sky were beaming like the stars in the Ripple Experience.	Yes
3	4	No				
3	5	Yes	I was dreaming about me riding my new car but my car was also a bike at the same time. I was driving the speed limit of 30 then I was coming to a light but at the light it was also side parking. As I was approaching a party bus in front of me I couldn't press down on the breaks hard enough and my car slowly came to a fully stop as I was approaching the other vehicle but at the same time I tapped the other vehicle moving the party bus up about 4 yards.	No		Yes
3	6	Yes	Last night I had a dream about a young lady I follow, she is very inspirational and this dream was mathematical and I was in high school. We had to problem solve some math equations. Both of us was dresses nice and I was never good in solving math problems and she said I needed to find the "adverb" to solve the math problem.	No		Unsure
3	7	No				
3	8	Yes	So last night my strongest dream was me sleeping inside the dream Capsule room and thier were sleep scientists watching and observing me. The wall with the led lights¸ had windows and I could see people outside. It look like a dark forest and people camping and camp back packs.	Yes	When I was sleep last night inside the "Dream Capsule" my dream looked like the "Ripple Experience " with the stars and music in the background. I did have the "Ripple Experience " in my dream last night and that felt good. The dream Capsule look like the Experience I had with my partner in the VR.	Yes
3	9	No				
3	10	Yes	Last night the dream I remembered the strongest is I was in my old neighborhood as a child and each home on the block had their own Nebula (cloud) over thier home. Each home Nebula had a different color and it was also a video camera recoding what's going on outside the home.	Yes	The Colorful Nebula Clouds represented us in the VR Ripple Experience. I the Ripple Experience my Cloud¸ (myself) is purple and my partner is orange.	No
3	11	No				
3	12	Yes	Last night I had dream about a little frog in my shoe. The frog would hide in a deep part of my shoe sole every time I start walking around somewhere. The frog eventually had a family inside my shoe with 2 children and a wife.	No		Unsure
3	13	No				
3	14	No				
4	1	Yes	I was back in the production of The Dolphin Show Kinky Boots showing the child actor of Lola his props and where to put them. He kept hiding from me and running around.	No		No
4	2	Yes	I was on a mattress driven by a horse and my alchemy pack with all my plant ingredients kept spilling out behind us :(.	No		No
4	3	No				
4	4	Yes	I am babysitting 2-3 kids and I leave them with my cousin to go get an ICEE. Halfway there I realize I am dreaming and control how fast/where I am going.	No		Yes
4	5	Yes	I was part of a system where 2 people (strangers) checked in with another person before going into a room with classic activities (drinks, arcade, etc.) to try and connect without knowing each other, like a blind date. I got a peach drink and the person won me a peach stuffed animal.	Yes	Going into a separate space from everyone else and being encouraged to connect with a stranger	Yes
4	6	Yes	I was at high school and looking for flowers I left in my locker to give to some old ladies.	No		No
4	7	Yes	I was getting strawberries from my high school's cafeteria and yelled at a worker for being mean to a low income student.	No		No
4	8	Yes	I am opening a door in my apartment and trying to open it to a door in my house in MN, but there's just more doors.	No		Yes
4	9	Yes	I was fishing with my hand??? And cleaning a shower.	No		No
4	10	Yes	I was in high school again and everyone said they hated me (rude).	No		No
4	11	Yes	I worked at a vet's office washing dogs and my grandmother shot me.	No		No
4	12	Yes	I was in the universe of guardians of gahoole.	No		No
4	13	Yes	I was clearing up hair clips and string from my childhood so I could help vacuum and clean up our family house.	No		Yes
4	14	Yes	I was flying with my brother and we had pet pokemon.	No		Yes
4	15	Yes	I was at my church with TikTok cosplayers planning a challenge to throw together cosplays in one hour.	No		Yes
4	16	Yes	I had a night terror where I couldn't move but a shadow creature looked over me and scared me.	No		Yes
4	17	Yes	Three of my old friends and my mom all sat down with me and talked about ways I could improve.	Yes	It was a heartfelt moment where we all sat down like the experience	No
4	18	Yes	I was at church with my family and was trying to show them that I could fly, but when I did it, only my brother believed me and the rest of my family didn't perceive it and thought I was just standing there.	No		No
4	19	Yes	I was spending time with my dead friend.	No		No
4	20	Yes	I had to bend over and put my back against a wall while still standing up and soon a hula hoop with my arms.	No		No
4	21	No				
4	22	Yes	I beat up my brother :(	No		No

At-Home Dream Report Coded Evaluation Grid

Participant	Dream Report Number	Lucidity	Volitional control	Awe	Lab incorporation.	Sniffing signals	LRLR Eye signaling	Other experience	VR: energetic body (blobs of light, balls of light)	VR: light streams (seeing streams of light)	VR: darkness
1	1										
1	2										
2	1							Feelings evoked questioning sense of self/ego			
2	2										
2	3				Yes (EEG)						
2	4										
2	5	Yes									
2	6										
2	7	Yes	Yes, could fly								
2	8										
2	9										
2	10										
2	11							Reuniting			
2	12				Yes (Lab tests)						
2	13										
2	14										
2	15										
2	16										
2	17										
2	18										
2	19							Intentional breathing	Yes		
2	20										
2	21										
2	22										
2	23										
2	24							Telepathy			
2	25										
3	1								Yes (colored smoke)		
3	2										
3	3							Being in a powerful body			
3	4										
3	5										
3	6										
3	7										
3	8	Yes	Yes		Yes	Yes	Yes	"I felt so at peace and good"	Yes		Yes
3	9										
3	10				No				Yes (nebula clouds in colors)		
3	11										
3	12										
3	13										
3	14										
4	1										
4	2										
4	3										
4	4	Yes	Yes								
4	5							Connecting with stranger			
4	6										
4	7										
4	8										
4	9										
4	10										
4	11										
4	12										
4	13										
4	14										
4	15										
4	16										
4	17							Connecting with loved ones			
4	18										
4	19							Connecting with deceased			
4	20										
4	21										
4	22										

Final Debriefing Interview Questions:

1. How was your experience in the study?
2. Did the Ripple experience change between your first and second session?
3. How did the overnight experience impact your perception of the Ripple experience?
4. Did your relationship to your dreams change from week one to week two?
5. Was there any part of participation in the study that resonated with you the most?
6. Did you experience any unexpected emotions during Ripple?
7. Did you have any lucid dreams? If so, did they connect with any aspects of the Ripple experience?
8. Would you consider using the Ripple experience at home for your own goals or interest?
9. Has the Ripple experience, dream journaling, or in-lab experiences had an effect on your waking life?
10. Do you have any other feedback?

Final Interview Quotations

Participant 2

"I remember being very shocked when the experience said to give everything you have, such as your best qualities, to the other person, everything. Then I remember feeling, okay. I'm going to give everything. But what am I going to stay with? I felt resistance about giving. Right? I was like okay... then who am I?"

"… my brain started giving me sensations… the experience of *Ripple* was fooling my brain. The sensations and even smells. At some point, I was having smells. I was feeling it in my body. I was very immersed. I was like, 'this is crazy! This is not happening!' but my brain already recon-structed the smell, reconstructed the feeling when we were merging in *Ripple*. It's like I could feel it in my skin. It was like how the [energetic bodies] were coming together. I was like, 'This is just insane'. And then that carried over into my week where I was like, this is so powerful on how you can fool your brain to believe the things that happened. So wow."

"When (I) went to sleep in the lab, It just made it more dreamy. Right? Like, I think the *Ripple* is already, like, a very dreamy experience, and you are, like, in the space and the music, and I think it's very, very well chosen. I love this project because of that. It just brought me such a comfort, a sensation of comfort, and the music. I just felt loved and it was very nice. I felt a lot of love. *Ripple* also was made with so much care, consideration, and compassion for people, which is something that I personally, like, really appreciate too."

Participant 3

"I realized I was dreaming. So that was when I became lucid in my dream, and that was when I did the eye signal. I That was when I did my eye signal of looking left, right, left, right twice." "It was dark, but the room looked longer for some reason, I was in the bed. And the gas, it was going in and out, in and out. It did it about 4 times, and then it got dark and the music came on. And that was when I realized it was over. So I sniffed 4 times. The manual said, once it's over, you sniff 4 times. Music kinda came in and and it got dark, so I didn't see the clouds no more.

Participant 4

"I liked when our bodies changed into the same color… we spent time doing the same thing, joining together, and being "one", and becoming the same thing. It was interesting, especially since we already didn't have physical bodies. The things that represent our physical bodies were reflections of each other. Almost like an erasure of differences or divisions."

"I had 2 dreams. I remember one of them, I was talking to my mom and, like, flying off into space, which I found to be connected to the *Ripple* experience just because, like, it's in space, and I was focusing on the the moment in the experience where all the light goes into darkness. I don't remember the other dream, but I forgot.

"I was in a car, flying up into space with my friend, but my friend turned into my mom. And we held hands and reconciled because earlier before the study, I had a fight with my mom."

Participant 3 drawing of a dream

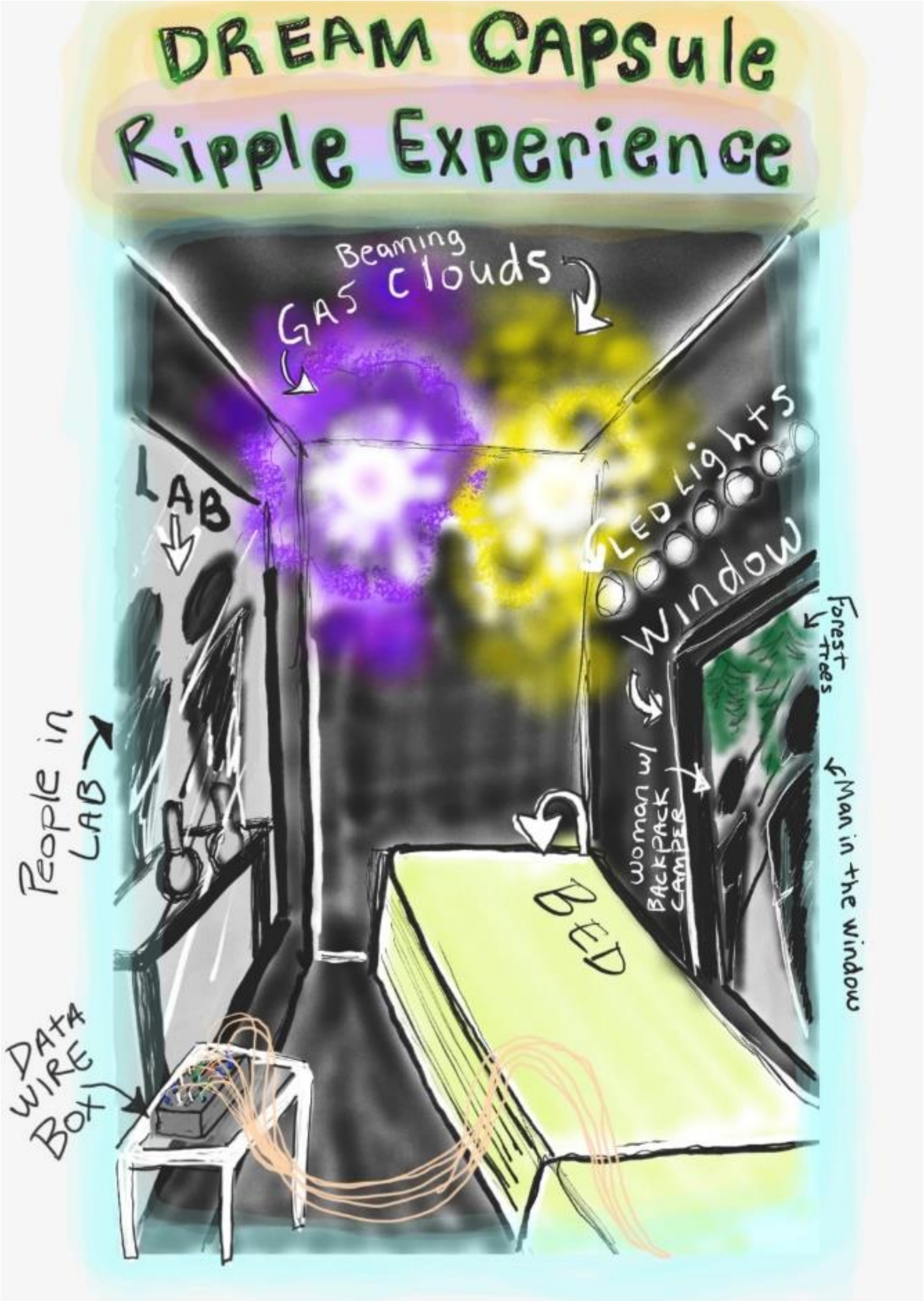

Participant 3 micro-phenomenology analysis

<table>
<tr><th>Summary of phase</th><th>Illustrative interview excerpts</th></tr>
<tr><th colspan="2">Phase 1: Just observing / Praying mantis (Non-lucid dream)</th></tr>
<tr><td> Context (whole experience): The experience starts in the lab room (“dream capsule”), with the dreamer on her back and looking up towards the wall in front of her, in half sitting, half laying position. The room or the perspective on the room is elongated. It is mostly dark but there is a little bit of light coming from the LED lights (“stars” from the initial dream report in the lab). The perceived physical environment stays the same throughout the experience; the only thing that changes is the perspective and body position, and different elements of the room entering into and leaving the experience accordingly. The experience begins with the dreamer observing the wall that she's facing with her eyes open. Her point of view is as if she was seeing from her eyes, but more panoramic or wide. She is feeling quiet, still, there is no sound or movement; she is staring without blinking, only moving her gaze around the visual field. She describes the way in which she is observing and paying attention with the metaphors “like a child” and “like a praying mantis”. She is feeling comfortable, nice, at peace. She doesn't recall having any thoughts or experiencing any bodily sensations, although she says that she felt like she was in her body. In terms of meta-awareness and insight, this is a non-lucid phase; the dreamer feels like she is awake and she is aware that she is in the lab room and currently participating in the experiment. This awareness of participant in the experiment remains present throughout the experience. </td><td> It's a very quiet moment. It's very still. It looks just like I'm awake. … And I was just looking, just observing. [The wall] looked a little further away … It looked in a perspective way. … Like I had a more, it was a longer vision. I don't know what it feels like to be a praying mantis … when I observe one, they look like they're at peace … I was just so comfortable and just sitting there looking, I wasn't bored, I felt a sense of energy from it. … It felt good just to sit there. I feel like a child … a child would just sit and observe. … I know I'm moving my eyes. I'm observing, looking at everything around me. It feels so good. It was just straight keeping my eyes open. … Still, straight stillness. … No sound. No movement, no lights, blinking or dimming. It was silence. It was just like you was captured in time. </td></tr>
<tr><th colspan="2">Phase 2: Window to a forest: Observing → Being observed (Non-lucid dream, “spark of lucidity”)</th></tr>
<tr><td> Unclear transition from the previous phase; some inconsistency in the description regarding whether it comes straight after. The dreamer is on her right side but still in half sitting, half laying down position. She is looking through the window on her right through which she can see the forest and hikers with backpacks who seemed to be getting ready to go somewhere; it is dark outside. As soon as the window enters her awareness, she notices that “something is different”, but explains these elements away as a part of the experiment setting. In the interview, she describes this noticing as a “spark of lucidity”, but not full lucidity questioning; there is also no sense of being shocked or surprised. There are two subphases: in the first one, she is observing people through the window and noticing things; in the second one, a man comes towards the window and leans on it, observing her in a predatory way while eating fast food from a bag. She is nevertheless feeling safe and protected by the room. </td><td> I didn't know that [the window] existed until I turned and seen it. … but when I seen it, I wasn't shocked. … I still feel calm. … I’m thinking, ‘Okay, maybe this is part of the project. Maybe they put the lab in the forest.’ … I thought it was just part of [the experiment]… I didn't feel nothing wasn't normal. But I knew something was different. Even though I wasn't lucid yet, I knew something was different. … I said to myself … hmm they got a window here and … there's people outside. … I still felt that peace. I was just looking and I was like, oh, this is part of it. A guy was in the window eating fast food, looking at me. … observing me, but I felt like I was safe, ‘cause he’s outside… He leaned on the window, and he was eating out of this paper bag ... He was looking at me in a predator way. But I wasn’t scared - I knew he couldn’t get me. </td></tr>
</table>

**Phase 3: Rocking, seeing researchers / lab equipment (Non-lucid dream with pre-lucid / active questioning)**	
The next phase begins as she experiences rolling from the right side to the left side, in a similar half sitting, half laying position. The movement of rolling over to the other side into a movement of slowly rocking up and down, which she does four times, each cycle of going down and up taking around one minute in her experience.<br><br>As she faces the left side of the room on the first “rock”, she sees **Daniel next to her arranging the electrodes on the box on her left** (the “data wire box” in the sketch), focusing on his hands. On the second “rock”, she notices the **lab room through the door on the left and several researchers** working in white lab coats.<br><br>**<u>Transition: Onset of lucidity:</u>** During the second rocking movement she realizes that she is dreaming. She describes that **what made her realize that she was dreaming was the rocking**, because this was the only time that **she was moving** in the whole experience, and because the **rocking was so slow, and she was asking herself how that was possible**. Noticing the lab people seems to have been relevant for the lucidity onset, but she did not mention that connection explicitly. She describes the realization that she is dreaming **as a verbalized thought that she internally said to herself “in her head”: “I’m dreaming!”**.<br><br>The realization is immediately followed by what she describes as **alert excitement** and **focus on the tasks**: she does the **left right left right eye movement**, during which her visual focus stays on the lab people on the left, but at the same time she also experiences her eyes as being closed. She continues rocking throughout this sequence.	*I was doing this **slow rocking**. It felt like it took me a whole minute to get from here [shows lying down on right side] over to here [shows lying down on left side].*<br><br>*Daniel, I don't remember him opening the door, I just remember him doing like this [shows hand gesture] with that data box with the wires. … His hands doing like this and the door open. … I'm just laying down, just letting him fix the electrodes … I may have knocked the electrode off and I just thought he was just coming to fix it.*<br><br>*[Becoming lucid] is a gradual process. It's not like, boom. … After Daniel finished doing his thing, and I started the second rock, **I noticed those people was in the lab**, and I was like, oh, I knew … [But] **the rock was the thing that made me realize that I was lucid** ... Because it felt like I was moving, that's the only time I felt like I was moving, up and down, up and down. It woke me up to think in my head: I think you dreaming now. …*<br><br>*It **felt like I was falling but real slow**, and I was like, ‘Why is it taking so long?’ … I said … in my head, **why is it taking me a whole minute to rock**? Oh… Realizing, you are lucid now, you are becoming lucid. … **I said what? I’m dreaming! You say it in your head real loud: I’m dreaming**.*<br><br>*It was like the rush like, wow, I'm lucid! … Like **alert exciting**. Cause I had to remind myself to do these things … I was kind of going out of me, but I was hoping that I would still sleep, then you could get the data. … And I did all the cues so then you know of when I became lucid. … That was **when I did the eye movement** and then I went down and up. Down and up. ... While I was doing that, **I was still looking at the people**, they were still there.*<br><br>*The room, **everything gets a little bit brighter** because it is like an enlightening … so when you become more awakened … the room got a little bit more brighter. …*
**Phase 4: Preparing for the ripple experience to happen (Lucid dream)**	
She keeps rocking and looking at the lab on the left while turned left, while already expecting the ripple experience to happen. She describes that **she didn't know when the experience was going to happen but she knew that it would happen**, as this was part of the experiment: she knew it in a similar way that she knew that she had to do the eye movement after becoming lucid, and that she was supposed to do the sniffing signals when the ripple appears.<br><br>In the interview, she clarifies that it felt like the expectation was “implanted in her head”, and recalls that when giving her instructions, the experimenter didn't say “**if** the ripple happens” but “**when** the ripple happens”.	*I didn't know [when the ripple will happen] – but I know … **Something told me that I need to just look and allow it to happen**. … And how do we allow things to happen is by **being still** … I didn't know WHEN it was going to happen, but **I was expecting it to happen** … Because you guys gave me an assignment.*<br><br>*When I realized I was lucid, I was following what Daniel said: when you experienced the ripple effect to get the cue. … He said in that order: when you realize you are lucid, you do the eye movement. … Then when you start to see the ripple effect, you let me know by you doing the two sniffs. … And when the ripple is over, you do the four sniffs. … All of that sinks in.*<br><br>*It got recorded in my head that was supposed to happen. … He didn't say … IF you see it, he said WHEN you see it, when it starts, this is what you do. … So it was already put into motion that it was gonna happen. I did not know when. … **I prepared myself to look around and observe when it was going to happen**.*

**Phase 5: Ripple experience (Lucid dream with target experience)**	
After four full rocks, the participant knew that that was enough rocking and she ends up laying still on her back looking at the ceiling / wall in front of her like in Phase 1. She has been **waiting for the ripple experience to happen** and describes **that she “needed to allow it to happen”**.<br><br>She **notices the two clouds that are illuminating and fading, as if breathing in and out, in purple and orange color**, respectively. It feels like the clouds were already formed earlier but that she is just noticing them now. She immediately does the two sniffs signal, as this is the ripple experience.<br><br>During this experience **her attention is totally focused on the two clouds**; she describes this as **“tunnel vision”**, and explains that this part of the experience was so special that she wouldn't want to look at anything else.<br><br>The embodiment and sense of body seems to be different in this phase. The participant says that she didn't feel like in her body, but felt more like a floating head. She **feels light, airy, “like you could float and move”**, there seems to be a **decrease in the sense of boundary** which she describes as **“like things could come through you”**.<br><br>She describes a **very positive affective experience** with strong elements of **awe** (although she herself doesn't use that word). Seeing the illuminating clouds **feels like being connected to them**, she feels a **sense of calm but all-encompassing excitement**, she feels **accepted and like she belongs**, and also that **it is very important to witness this experience**.	*Something just told me to stop [rocking]. And I did. … I remember the transition from doing like that [showing - being on her left side] to like this [showing - being on her back]. That was when … I started seeing them.*<br><br>*It was in that gassy cloud. … It looked just like me and Blaise when I was in the virtual reality thing. … I just watched. I allow it to do like this [pulsing gesture], the clouds to go light, to get dimmed, and go in and out. … Illuminating in and out.*<br><br>*It's just like they was breathing … They sent the signal of what I experienced in the VR, I was just waiting to see if it was similar … That's the only way I knew that that was the experience [i.e. the ripple experience].*<br><br>*The gas, the clouds, they was there. … they started to get bright. And then they go like this, dark... And then bright. So I'm looking and I'm like, wow, I'm actually experiencing it. That was when I did the cue of the sniffs, to let you know that I'm in that moment. … It seemed like I was in that moment for a little while. It seemed like it was some good two minutes. That's a long time. … It went about three or four times, and then after that it started to get dark and the music came on.*<br><br>*It was stars everywhere. … It was very neon colors. And it was glowing. … The lights on the wall, those was the stars. I didn't realize they was there until I realized I was lucid and then they started to get bright.*<br><br>*My focus, I had tunnel vision, straight on the clouds. … It was straight focus on that. It was so beautiful and such a great experience. … I didn't want to look at nothing else. … It's like I get, I get to see this. That's why I felt special: I get to see this. … It was a calmness. It was a more excited calmness though.*<br><br>*I was just looking up at it and it just felt so good. … It's like a silent communication. And I was looking up at it, and I swear it looked just like what I doodled. … I was paying full attention to that.*<br><br>*That's how everything started [in Phase 1] … I was just observant. … But with the ripple effect … I'm looking, I'm still observant. … I'm seeing movements of light, and I'm feeling that onto my face. And I'm still feeling like the way I was feeling [i.e., observant], but it was a little different because I'm lucid now, and I feel like I was having an interaction with what I was seeing.*<br><br>*I’m looking for answers … by just focusing on the light. … I'm looking in and I’m searching for light. … It's like something in me knows to do it. It’s something in me that knows: this is what I'm supposed to be doing. This is what I'm supposed to be looking at. … It just felt like: that was how I was gonna get my answers.*<br><br>*I'm waiting to receive some answers. … And I'm just looking in the light and it's just beaming on me. … It's an inner feeling. It doesn't have expression. … It's a feeling of something that you can't communicate by words.*<br><br>*I don't even really feel like I was in my body. I would feel like I was a part of that, like it was like a mirror, like a belief in mirror reflection. … I felt airy. … Like things could come through you, like you're floating, like you're lighter, I felt light. I felt connected. I felt like I could float and move... I feel soft.*<br><br>*Everything seemed like it was happening like this [gesturing in the space around her face]. It seemed my head was really floating. … I had like a floating head. <Laugh>. It didn't feel like I was sitting or laying down, but I was positioned.*<br><br>*I felt accepted. … I felt I belong. … It wasn't no fear, it felt really good. It was, it was amazing, just, just to see. I felt, I feel beauty, you know, it was beauty that in that moment of the colors.*

<table>
<tr><td>There is a strong sense of meaning in the experience it feels like the two clouds are communicating with her, beaming energy to her that is the same kind of energy that she was giving in the VR experience. She describes the experience of waiting to receive answers, she feels like she's looking for answers related to her past and her childhood by focusing on the light, which feels like the thing she is supposed to be doing. Receiving energy from the clouds as they breathe out is experienced like feeling warmth, heat, or sunlight on and a bit outside of her face.</td><td>They're still where they're at, but … illuminating in and out, they wasn't floating around … Like it's just like they was breathing like [shows: inhale] when they breathe in, they close when they breathe out. But when they breathe out, that's when I feel the HEAT onto my face.

I don't know if they was breathing. But it SEEMED like they would. … It was just like what I did in the VR exercise: When you breathe in, it's tight. When you breathe out, that's when they beam open. So, they [the clouds] was blowing towards ME, so I could receive the energy. … That's what feels like the CONNECTION. … And that to me was a mirror image of what I was doing in the VR. … I was giving it that same energy, so the dream was giving it to me back.

[In the VR] I was the purple cloud, but now I could see the clouds [laughs].

I felt enlightened. It felt so good. I don't even know if I felt like I was in, in my human body. I felt like I was like a part of that experience of what it is I was seeing. … It almost makes you wanna cry. ... I felt like the light was communicating to me in the dream. I felt, I knew it was the ripple, and that's only because of what I experienced [earlier].

It felt like warmth … located on my face, up in here [showing]. It kind of feels like a sunlight hitting. Beaming on you. … It's hard to explain. … I'm observing, but I'm also taken in. … It was, just like, if you was to take your hand and put it next to your face, you could feel the warmth on your hands ... I felt like it was a presence there.

I wanted to experience as much as I could, and see as much as I could, cause my job, my duty, my goal was to stare at it and see it as much as I could see it. It's just like looking at an eclipse. You know how it only lasts for a little while? It's that same feeling that that's special to see that. … I'm just looking and it's happening and I'm wanting this moment, this moment is very important. I know that I have to see this moment. And I felt good and I felt happy and I felt deserving and expecting. … And I knew that I had this mission that I had to see it. It was so important to me.

Everything seemed like it was happening like this [gesturing in the space around her face]. It seemed my head was really floating. … I had like a floating head [laughs]. It didn't feel like I was sitting or laying down, but I was positioned [in space]. … You supposed to just take my eyeballs and just put them in the room, because I was just eyeballs.</td></tr>
<tr><th colspan="2">Phase 6: Clouds getting darker, music coming in (Lucid dream) → Being woken up</th></tr>
<tr><td>The ripple experience ends by the clouds getting dark after which the music comes in. In the interview, she says that this is the first time she remembers hearing music (or any sounds) in the whole experience – mismatched with initial dream report. It feels like the music is coming from her right side and it feels very nice.

She knows that the ripple experience is over and does the four sniffs to signal that. She is feeling neutral, not sad, and is soon woken up by Daniel, although she would have liked to stay in that state.

The experience after the four sniffs and before being woken up was not explored in detail because the participant didn't seem very confident in her memory. However, the experience seems to have remained lucid until the end.</td><td>And then when the music came along … I don't know if the music was playing all along. I just know that I heard it before the dream was over. It got a little dark, for like a good 15, 10 seconds, and then when the music came on … It was coming from my right side … And I said, it is over with, and then that was when he woke me up.</td></tr>
</table>

Note about the mismatch between the interview and the dream report – from a conversation at the end of the second interview

Interviewer (01:56:16): One more question because I did see your dream report that you gave in the lab, right after Daniel woke you up, you were talking about electrical beings and mechanisms and aliens…

Participant (01:56:40): Oh yeah. So I was talking about this movie. It was called *Batteries Not Included*. And that's one of my favorite movies. Cause it's these drones that these robots built and they come from this other planet. … It came out in the early eighties. And they fly around and them clouds was reminding me of *Batteries Not Included*. … That was my association… It was reminding me of that movie because the cloud was floating in the air, just like the robot drone was. I was making that similarity. Cause you know, it's like, the Ripple experience is still kind of spiritually alien. I feel like it is like the midpoint of Alien and Human. … It did, it did feel like that, you know?

In the interviews, the participant explained that some elements of the initial lab report (e.g., "floating computers," "aliens," "jellyfish") were associative stand-in metaphors produced in the confusion of awakening to convey the "alien" atmosphere of the dream, which reminded her of the film Batteries Not Included, rather than descriptions of the actual elements of her dream.
